# Enhancement of Frequency Domain Indices of Heart Rate Variability by Cholinergic Stimulation with Pyridostigmine Bromide

**Published:** 2011

**Authors:** Ali Asghar Zarei, Seyyed Abbas Foroutan, Seyyed Mohsen Foroutan, Abbas Erfanian Omidvar

**Affiliations:** a *Department of Physiology, School of Medicine, Shahid Beheshti University of Medical Sciences, Tehran, Iran.*; b *Neuroscience Research Center*, *Shahid Beheshti University of Medical Sciences*, *Tehran, Iran.*; c *Department of Pharmaceutics, School of Pharmacy, Shahid Beheshti University of Medical Sciences , Tehran, Iran.*; d *Department of Medical Engineering , School of Electrical Engineering , Iran University of Science and Technology, Tehran, Iran.*

**Keywords:** Pyridostigmine bromide, Heart rate variability, Cholinergic stimulation, Cholinesterase inhibitors

## Abstract

Pyridostigmine bromide (PB) is a reversible cholinesterase inhibitor. The aim of this study was to determine the effect of orally administration of single dose sustained-released tablet of pyridostigmine bromide (PBSR) on the frequency domain indices of heart rate variability (HRV). Thirty-two healthy young men were participated in this study. They were divided into 2 groups; the pyridostigmine group (n = 22) and the placebo group (n = 10). Electrocardiogram (ECG) was recorded at 10, 30, 60, 90, 120, 150, 180, 210, 240, 300 and 420 min after PBSR administration. At each time, simultaneously, a blood sample was prepared and PB plasma concentration was measured by high-performance liquid chromatography (HPLC) method.

Statistical analysis showed that in different indices of HRV, there is a significant increase in low frequency (LF) band at 300 min, but no difference in high frequency band (HF). It also showed significant decreases in normalized high frequency band (Hfnu), normalized low frequency band (Lfnu) and LF/HF ratio at 120, 240 and 300 min after PBSR administration. Maximum plasma concentration of PB was 150 min after the administration. In conclusion, administration of a single dose PBSR can enhance the frequency domains indices of HRV and improvesympathovagal balance.

## Introduction

Heart rate variability (HRV) is a physiological phenomenon where the time interval between heart beats varies. Reduced HRV has been shown to be a predictor of mortality after myocardial infarction (1, 2).

Pyridostigmine bromide (PB), a reversible cholinesterase inhibitor, is used as an antagonist for treatment of myasthenia gravis (3).

Cholinergic stimulation with PB in patient with a 24 h heart failure increase duration between two consecutive R waves (R-R interval) and long-term time domain indices of HRV (4) and also in healthy individuals (5). A number of studies have indicated that PB decreases the resting heart rate in comparison with placebo in sedentary subjects but does not significantly change it in trained athletes whereas time and frequency domain indices of HRV do not differ after PB administration versus placebo in sedentary subjects or trained athletes (6). Other studies have also signified that PB slows the heart rate and decrease the high frequency band (HF) of HRV (7, 8). Animal studies have shown improvement in the time and frequency domain indices of HRV after 7 days administration in conscious and unrestrained rats (9). *The present investigation was carried out to explore *the effect of orally administration of single dose 180 mg sustain-released tablet of PB (PBSR) on the frequency domain indices of HRV.

**Figure 1:**
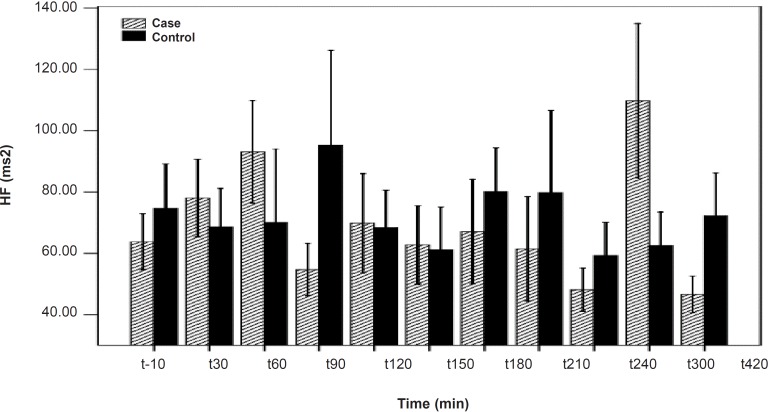
HF differences between the PB and placebo groups. No significant difference was seen between the PB and placebo groups. Data are shown as mean ± SD

## Experimental

*Subjects*

Thirty-two healthy non-smoking male adult volunteers participated in this study. All the volunteers gave written informed consent after explaining the nature and purpose of this study and possible adverse effects. Subjects were free from significant cardiac, hepatic, renal, pulmonary, neurological, gastrointestinal and any acute or chronic disease as determined from their medical history, clinical examination, and laboratory investigation (hematology, blood chemistry and urine analysis). The volunteers were asked to abstain from taking any medicine, for at least two weeks prior to and during the study. They were randomly divided into two groups; the pyridostigmine group (n = 22 ; mean age was 23.86 ± 2.69) and the placebo group (n = 10 ; mean age was 24.5 ± 3.43). Each subject was fasted overnight prior to the experiment and food was withheld for 3 h after dosing. A venous canula was inserted in the volunteers› antecubital vein. A 5 min ECG recorded 10 min before PBSR administration by Power Lab set model 4/25T and a blood sample collected. Then, a single dose of 180 mg PBSR tablet and placebo were prescribed to the pyridostigmine group and placebo group, respectively. This dose of PB was very well tolerated. Afterward, ECG was recorded at 30, 60, 90, 120, 150, 180, 210, 240, 300 and 420 min after the PB administration. At each time, a blood sample was collected simultaneously from venous canula. Blood samples were transferred to the laboratory immediately and centrifuged by 4000 rpm. Plasma was extracted from blood and stored at -20°C. When all the plasma samples were collected, PB concentrations in the plasma were measured using high-performance liquid chromatography (HPLC) method. HRV was analyzed by MATLAB software version 7.6a. Bessel distribution (one of the HRV analyzing methods) modified by the Bioelectric Department of Science and Technology University of Iran was selected for data analysis. 

*Statistical analysis*

Data was analyzed statistically using independent t-test; when data distribution was not normal, Mann-Whitney test was used instead. SPSS software version-16 was used for the statistical analysis. Results of both pyridostigmine and placebo groups were compared with each other before and after the PBSR prescription.

**Figure 2:**
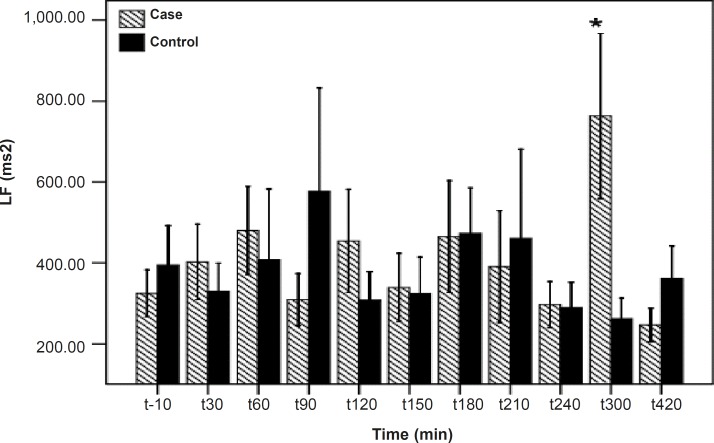
LF differences between the PB and placebo groups. There is a significant difference between the PB and placebo groups at 300 min after the PBSR administration. Data are shown as mean ± SD

**Figure 3:**
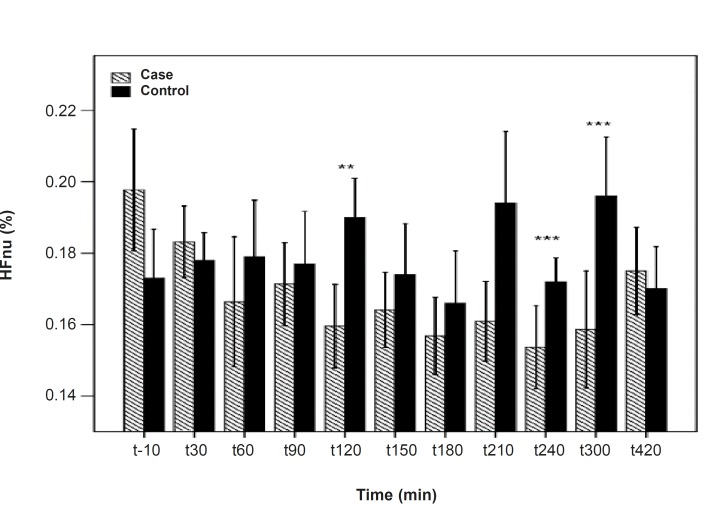
HFnu differences between the PB and placebo groups. There are significant differences between the PB and placebo groups at 120, 240 and 300 min after the PBSR administration. Data are shown as mean ± SD

**Figure 4:**
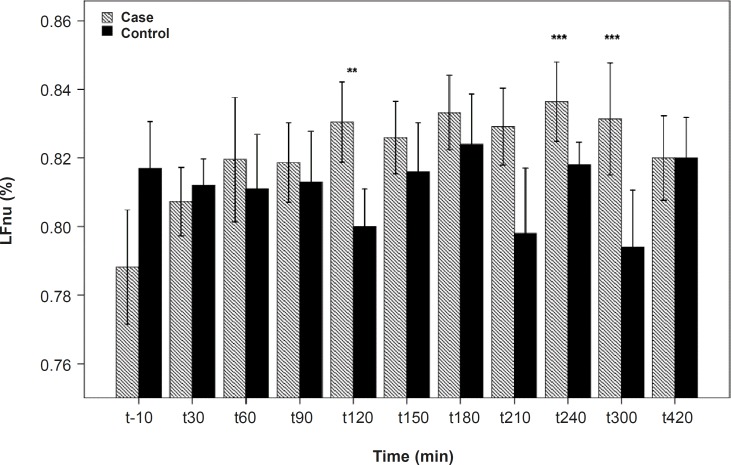
LFnu differences between the PB and placebo groups. There are significant differences between the PB and placebo groups at 120, 240 and 300 min after the PBSR administration. Data are shown as mean ± SD

**Figure 5:**
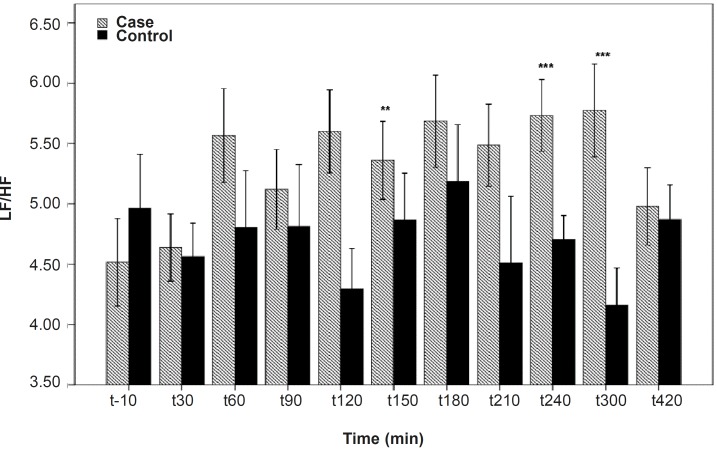
LF/HF differences between the PB and placebo groups. Significant differences were seen between the PB and placebo groups at 120, 240 and 300 min after the PB administration. Data are shown as mean ± SD

**Figure 6:**
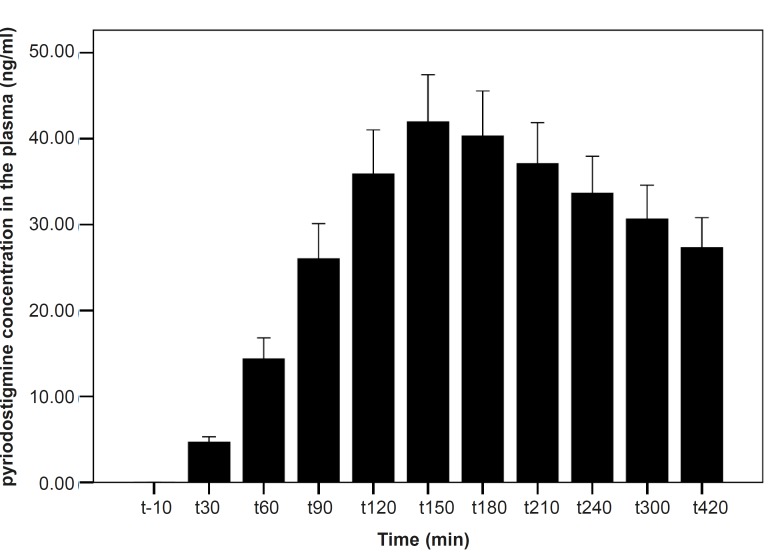
PB concentration in plasma for the PB group. PB concentration reached the peak at 150 min after the PBSR administration. Data are shown as mean ± SD

## Results and Discussion

As shown in Table 1, after the pyridostigmine administration, there are no significant changes in high frequency band (HF), a significant increase in low frequency band (LF) at 300 min after the PB administration, significant decreases in normalized high frequency band (Hfnu) at 120, 240 and 300 min after the PB administration. There are significant increases in normalized low frequency band (Lfnu) at 120, 240 and 300 min after the PB administration and also significant increases in LF/HF at 120, 240 and 300 min after the PBSR administration. PB concentration in plasma measured by HPLC method reached the peak at 150 min after dosing. 

**Table 1:** Frequency domain indices of HRV before and after the PB administration

**Time**	**HF**	**LF**	**Hfnu**	**LFnu**	**LF/HF**
**-10**	0.52	0.52	0.47	0.32	0.37
**30**	0.65	0.62	0.93	0.91	0.87
**60**	0.44	0.72	0.17	0.26	0.22
**90**	0.23	0.33	0.58	0.58	0.48
**120**	0.95	0.46	0.01**	0.01**	0.009***
**150**	0.94	0.91	0.37	0.37	0.47
**180**	0.63	0.97	0.44	0.44	0.41
**210**	0.55	0.78	0.09	0.10	0.10
**240**	0.38	0.93	0.009***	0.009***	0.008***
**300**	0.14	0.02*	0.008***	0.008***	0.007***
**420**	0.055	0.16	0.41	0.23	0.46

p = p-value of compensation between the PB and placebo groups of related index. * 0.01 < p-value < 0.05; ** p-value = 0.01; *** p-value < 0.01

It was reported that PB can not alter the frequency domain indices of HRV (5, 6) but Cook *et al*. showed that PB increases LF and decreases HF which is dose-dependent and intensified through dose increasing (7). In the present study, similar to Cook›s study, increasing in LF and LF/HF was observed. An increase in LF becomes evident when it is shown in normal unit (LFnu) and also when is shown as LF/HF ratio (10). Some believe that Lfnu reflexes the sympathetic modulation and others consider LF as reflecting both sympathetic and vagal activity. It was also suggested that the LF/HF ratio is a better indicator compared to LF for showing the sympathovagal balance (10, 11).

PB cannot pass through blood-brain barrier; consequently, the effect of PB on frequency domain indices is peripheral and related to the increasing synaptic acetylcholine concentration (5). Acetylcholine acts on cardiac M_2 _receptors and diminish the heart rate (7) and thus, the baroreflex leads to increase sympathetic firing centrally (12) so, sympathetic activity tends to decrease the HF component and increase the LF.

*Our results indicate that *the most changes in the indices happened at 120, 240 and 300 min after the PBSR administration. These points of time are related to the PB concentration of 45-52 ng/mL, whereas, maximum PB concentration is 61.04 ± 14.07. Very low and very high «tonic» parasympathetic activities might result in actual decrease in HRV while the intermediate level might result in the strongest HRV (11). According to the present study, PB concentration of 45-52 ng/mL probably mimics an intermediate parasympathetic activity. 

## Conclusion

Administration of a single dose PBSR can enhance frequency domains indices of HRV and improve the sympathovagal balance. Further studies are needed to determine the effect of long-term administration of PBSR in healthy individuals and especially in patients with heart disease. 

## References

[1] Bigger JT, Fleiss JL, Steinman RC, Rolnitzky LM, Kleiger RE, Rottman JN (1992). Frequency domain measures of heart period variability and mortality after myocardial infarction. Circulation.

[2] Kleiger RE, Miller JP, Bigger JT Jr, Moss AJ (1987). Decreased heart rate variability and its association with increased mortality after acute myocardial infarction. Am. J. Cardiol.

[3] Berrouschot J, Baumann I, Kalischewski P, Sterker M, Schneider D (1997). Therapy of myasthenic crisis. Crit Care Med.

[4] Behling A, Moraes RS, Rohde LE, Ferlin EL, Nóbrega AC, Ribeiro JP (2003). Cholinergic stimulation with pyridostigmine reduces ventricular arrhythmia and enhances heart rate variability in heart failure. Am. Heart. J.

[5] Nóbrega AC, dos Reis AF, Moraes RS, Bastos BG, Ferlin EL, Ribeiro JP (2001). Enhancement of heart rate variability by cholinergic stimulation with pyridostigmine in healthy subjects. Clin. Auton. Res.

[6] Dewland TA, Androne AS, Lee FA, Lampert RJ, Katz SD (2007). Effect of acetylcholinesterase inhibition with pyridostigmine on cardiac parasympathetic function in sedentary adults and trained athletes. Am. J. Physiol. Heart. Circ. Physiol.

[7] Cook MR, Graham C, Sastre A, Gerkovich MM (2002). Physiological and performance effects of pyridostigmine bromide in healthy volunteers: a dose-response study. Psychopharmacology.

[8] Umeno S, Hori T, Ono S, Nishijo H (2004). EEGs and autonomic changes during and after acupuncture stimulation. Iranian J. Pharm. Res.

[9] Soares PP, da Nóbrega AC, Ushizima MR, Irigoyen MC (2004). Cholinergic stimulation with pyridostigmine increases heart rate variability and baroreflex sensitivity in rats. Auton. Neurosci.

[10] Heart rate variability: standards of measurement, physiological interpretation, clinical use (1996). Task Force of the European Society of Cardiology and the North American Society of Pacing and Electrophysiology. Circulation.

[11] Hedman A, Hartikainen J, Haukomaki M (1998). Physiological background underlying short-term heart rate variability. Ann. Noninvasive. Electrocardiol.

[12] Koeppen BM, Stanton BA (2008). Bern and Levy Physiology.

